# Optimum Control for Path Tracking Problem of Vehicle Handling Inverse Dynamics

**DOI:** 10.3390/s23156673

**Published:** 2023-07-25

**Authors:** Yingjie Liu, Dawei Cui, Wen Peng

**Affiliations:** 1 School of Machinery and Automation, Weifang University, Weifang 261061, China; wfxycdw@163.com; 2 State Key Laboratory of Rolling and Automation, Northeastern University, Shenyang 110819, China; pw3345@163.com

**Keywords:** vehicle dynamics, path tracking, optimal control, symplectic pseudospectral method

## Abstract

Accurate tracking of a given path is one of the primary factors in the maneuverability of a vehicle and is also an important topic in autonomous vehicle research. To solve the problem of vehicle path tracking, the problem must first be transformed into an optimal control problem. Then, a symplectic pseudospectral method (SPM) based on the third-generation function of symplectic theory and pseudospectral discretization is proposed to efficiently solve the nonlinear optimal control problems. Finally, the results obtained by the proposed algorithm are compared with those obtained by the Gauss pseudospectral method (GPM). The simulation results show that the proposed method can effectively solve the vehicle path tracking problem. Furthermore, the vehicle can track the given path controlled by the proposed algorithm with higher accuracy and greater applicability than other methods.

## 1. Introduction

Because of their convenience and comfort, vehicles are rapidly becoming indispensable to people’s lives. At this stage, global vehicle ownership is increasing annually. Additionally, it is difficult to decrease the incidence of traffic accidents because of the continuous increase in vehicle ownership. Traffic accidents are considered the “first public hazard” at present. The number of yearly traffic accident deaths has begun to exceed that caused by diseases and wars. As a key part of automated driving technology, vehicle motion control is at the end of the chain. Motion control captures the main behavior of the intelligent system and is the bridge between the vehicle and other modules. It plays an important role in supporting and realizing the algorithmic architecture of the auto-driving system. Based on the vehicle state and the planned trajectory, the motion control module controls the vehicle traveling along the planned path, including the speed, steering, and driving/braking systems. Obstacle avoidance path planning is an important technology in the field of automatic driving. It relies on various onboard systems to obtain road environment and self-driving information, using algorithms to plan a safe path so the vehicle can avoid collisions and bypass obstacles. The path tracking system ensures that the vehicle can accurately follow the expected path. Autonomous obstacle avoidance reduces injuries to passengers as well as economic and property losses caused by accidents. Furthermore, as the main interactor of intelligent transportation, it can alleviate the congestion caused by traffic accidents and reduce unnecessary losses. [1,2].

The problem of vehicle path tracking has been widely studied. A brief review is presented in the following.

Levy et al. [3] proposed a cooperative controller using a nonlinear model predictive control approach for dozens of autonomous vehicles without considering lane markings. In order to improve the accuracy of path tracking in intelligent vehicles, Shi et al. [4] proposed an intelligent vehicle path-tracking control method based on improved model predictive control combined with hybrid proportional-integral-derivative control theory. Awad et al. [5] introduced an integrated path-tracking control strategy for autonomous vehicles. To improve the path-tracking control performance of the intelligent vehicle under critical maneuvers, Sun et al. [6] introduced a novel control strategy. Liu et al. [7] presented the Gauss pseudospectral method (GPM) method to solve the vehicle path-tracking problem. Some researchers have focused on the nonlinear system but have not proposed further improving the solution’s accuracy.

Zhou et al. [8] pioneered the use of an adaptive inverse controller to offset the dynamics of the steering system’s backlash. Homolla et al. [9] proposed an encapsulated trajectory tracking controller. Hashemi et al. [10] presented a novel integrated control framework by considering the combined-slip effect, wheel dynamics, and tire force nonlinearities for driver-assistance systems. Fan et al. [11] proposed the synthesized safety distance for lane changing to study lane-changing time points. However, some models ignored the effect of kinematic changes on the tire. So related tire mechanics and the robustness of the algorithm should be studied.

Tan et al. [12] presented a robust model predictive control-based strategy for the path tracking of 4WS4WD vehicles considering external disturbances. Jiang et al. [13] proposed a hierarchical control strategy to realize the path-tracking control of six-wheel independent drive UGV based on differential steering. In order to overcome the structural and unstructured uncertainties, Jiang et al. [14] presented a model-free predictive control strategy using particle swarm optimization. However, most of the multi-degrees of freedom vehicle models were simplified. Therefore, a nonlinear model that can truly reflect the vehicle condition should be adopted.

In order to improve the path-tracking accuracy of the intelligent vehicle at high speed and establish a practical track-tracking controller, Shi et al. [15] established a vehicle monorail model to achieve the intelligent vehicle’s fifth-order polynomial obstacle avoidance path tracking based on the MPC algorithm. Vieira et al. [16] proposed an algorithm for path planning of car-like mobile robots without movement interruption and avoiding any obstacles along the way. Li et al. [17] designed a new obstacle avoidance path planner to solve the limitation of the traditional local obstacle avoidance path planner in excessive obstacle avoidance. However, most of them focused on the linear model, and they usually neglected the effect of solving methods from dynamic simulation. So, models that can truly reflect the vehicle motion condition and effective solving methods should be adopted.

Aiming to solve the problem of large solution errors and low accuracy, we first establish an optimal control model for trajectory planning. To achieve an efficient solution, under the indirect method′s framework and based on the third-generation function, high-precision pseudospectral interpolation is used for the first-order necessary conditions of the optimal control problem. Thus, a symplectic pseudospectral method is constructed. 

This paper proposes a Newton symplectic pseudospectral optimization algorithm, in conjunction with the Newton iterative method, to solve the vehicle path tracking problem. The remainder of the paper is organized as follows. Section 2 presents the problem of vehicle path tracking. Section 3 provides the Newton symplectic pseudospectral algorithm, and Section 4 presents numerical simulations and experimental verification. Finally, Section 5 concludes the paper and provides future research directions.

## 2. Mathematical Model of Vehicle Path-Tracking Problem

### 2.1. Mathematical Model of Vehicle Path Tracking Problem

**Figure 1:**
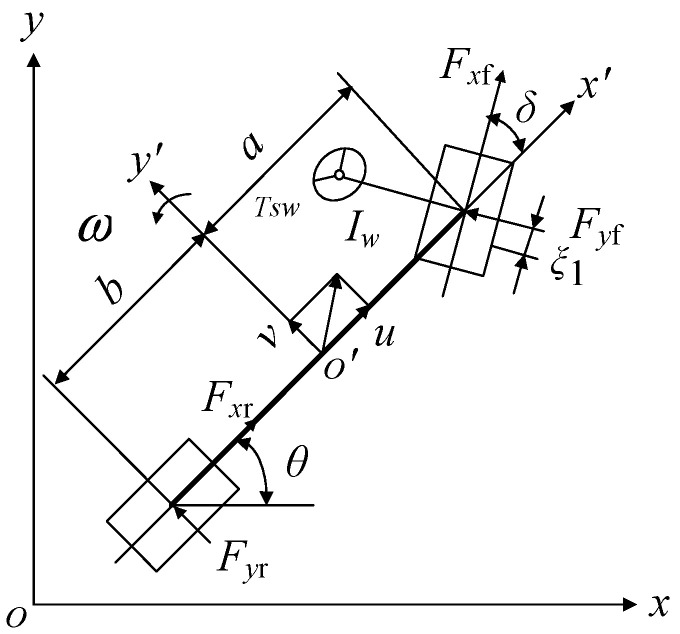
Nonlinear 7-DOF vehicle model.

The nonlinear 7-DOF vehicle model in Figure 1 describes the vehicle lane change problem.

In state–space form, it is [7]:

(1) $$\begin{array}{l} \dot{u}=v\omega +\frac{F_{xf}\cos\delta -F_{yf}\sin\delta +F_{xr}-F_{f}-F_{w}}{m}\\ \dot{v}=-u\omega +\frac{F_{yf}\cos\delta +F_{yr}+F_{xf}\sin\delta }{m}\\ \dot{\omega }=\frac{aF_{yf}\cos\delta -bF_{yr}+aF_{xf}\sin\delta }{I_{z}}\\ \dot{\delta }=p\\ \dot{p}=-\frac{k_{1}\xi_{1}}{I_{w}u}v-\frac{k_{1}\xi_{1}a}{I_{w}u}\omega +\frac{\left(k_{1}\xi_{1}-k_{w}\right)}{I_{w}}\delta -\frac{c_{w}}{I_{w}}p+\frac{T_{sw}i}{I_{w}}\\ \dot{x}=u\cos\theta -v\sin\theta \\ \dot{y}=v\cos\theta +u\sin\theta \end{array}$$

The state variable is $x(t)={\left[u(t),\;v(t)\;,\;r(t),\;x(t),\;y(t)\right]}^{T}$, and the control variable is $u(t)={\left[\dot{v}\right]}^{T}$. Then, Equation (2) can be obtained by simplifying Equation (1).

(2) $$\dot{x}=f[x(t),\;u(t)]$$

### 2.2. Optimal Control Object of Lane Change Motion Planning Problem

The vehicle path-tracking problem can be regarded as the optimal control problem in control theory. Therefore, the minimum lateral distance error of tracking the prescribed path is set as the control object:

(3) $$J=\int_{t_{0}}^{t_{f}}\left({\left(\frac{y-y_{d}}{\hat{E}}\right)}^{2}+{\left(\frac{z}{{\hat{T}}_{sw}}\right)}^{2}\right)dt=\int_{t_{0}}^{t_{f}}L(x,\;u,\;t)dt$$

where $t_{0}$ and $t_{f}$ are the initial and final time; $y_{d}$ is the desired path; $\hat{E}$ = 0.3 m is the standard threshold of the lateral distance error of $y-y_{d}$; ${\hat{T}}_{sw}$ = 8 N·m is the standard threshold of the steering torque.

### 2.3. Constraints

The initial and terminal states are described as:

(4) $$x(t_{0})={\left[u(t_{0})\;,\;0\;,\;0,\;0,\;0\;\right]}^{T}$$

(5) $$x(t_{f})={\left[u(t_{f})\;,\;0\;,\;0,\;x(t_{f})\;,\;y(t_{f})\;\right]}^{T}$$

When the braking maneuver is applied to decelerate the vehicle, the constraints on $F_{xf}$, $F_{xr}$ can be rewritten in the following manner:

(6) $$\begin{array}{l} F_{xf}\geq -\frac{\mu mg(b+\mu h_{g})}{a+b}\\ F_{xr}=\frac{a-\mu h_{g}}{b+\mu h_{g}}F_{xf} \end{array}\}$$

Therefore, the optimal path-tracking problem can be described as:

(7) $$\min J;\;s.t.\;\dot{x}=f[x(t),\;u(t),\;t],\;h\leq 0$$

where   h expresses the inequality constraint.

## 3. Newton Symplectic Pseudospectral Method

The terminal time and corresponding optimal trajectory are solved by the Newton symplectic pseudospectral method (NSP), which is based on the Newton iteration and the third kind of generating function as well as the symplectic theory, to obtain the optimal solution corresponding to the optimal trajectory. The specific calculation steps are:

**Step 1:** The final time in Equation (7) and the iteration accuracy of the Newton iteration method are initialized. The constrained time–energy optimal control problem in Equation (7) is transformed into an optimal control problem with constraints and a fixed final state and time using the quasi-linearization technique, which can be described as:

(8) $$\begin{array}{l} \;\min J^{\left[k+1\right]}=\frac{1}{2}\int_{t_{0}}^{t_{f}}({\left(U^{\left[k+1\right]}\right)}^{T}R^{\left[k+1\right]}U^{\left[k+1\right]})dt\\ s.t.\left\{ \begin{array}{l} {\dot{X}}^{\left[k+1\right]}=A{\;}^{\left[k\right]}X^{\left[k+1\right]}+B{\;}^{\left[k\right]}U^{\left[k+1\right]}+w{\;}^{\left[k\right]}\\ C{\;}^{\left[k\right]}X^{\left[k+1\right]}+D{\;}^{\left[k\right]}U^{\left[k+1\right]}+V{\;}^{\left[k\right]}+a{\;}^{\left[k\right]}\;=0 \end{array}\right. \end{array}$$

where  α is a non-negative compensation vector, and the number of state variables is set as $\;n_{s}$, $k=0,1,\cdots ,{\left(\cdot \right)}^{\left[k\right]}$ represents the calculation results of variables in the *k*th iteration. For convenience, superscripts indicating the number of iterations are omitted below.

For the above optimal control model, Lagrange operator  λ and parameter multiplier vector  β are introduced to unconstrain the original problem. The parameter multiplier vector satisfies $\;\alpha^{T}\beta =0,\;\beta \geq 0$. The objective function can be expressed as:

(9) $$\;J=\int_{t_{0}}^{t_{f}}(H-\lambda^{T}\dot{X})dt$$

where the Hamiltonian function is:

(10) $$H=\frac{1}{2}U^{T}RU+\lambda^{T}(AX+BU+W)+\beta^{T}(CX+DU+V+\alpha )$$

To minimize the objective function *J*, the system must satisfy both the control and Hamilton canonical equations. 

Then, the control equation is:

(11) $$\frac{\partial H}{\partial U}=UR+B^{T}\lambda +D^{T}\beta =0$$

The Hamilton canonical equation is:

(12) $$\dot{X}=\frac{\partial H}{\partial \lambda },\dot{\lambda }=-\frac{\partial H}{\partial X}$$

Since the Hamilton function is a function of state variables, control variables, Lagrange multiplier vectors, parameter multiplier vectors, and compensation vectors, and the control variable can be represented by the Lagrange multiplier vector and parameter multiplier vector according to Equation (11), the Hamilton function is a function of four independent variables: state variable, Lagrange multiplier vector, parameter multiplier vector, and compensation vector.

**Step 2:** Initializing the iteration parameters k=0, and setting the initial guess solutions of variables, control variables, and covariates as $X^{\left[0\right]}$, $U^{\left[0\right]}$, and $\lambda^{\left[0\right]}$, respectively.

**Step 3:** Combining $X^{\left[k\right]}$, $U^{\left[k\right]}$, and $\lambda^{\left[k\right]}$, the solution of Equation (8) is calculated by the third kind of symplectic pseudospectral method. The specific calculation steps follow.

The time interval $T=[t_{0},t_{f}]$ is discretized into *P* intervals. The *j*th interval is $T^{j}=[t_{j-1},t_{j}],j=1,2,\cdots ,P$. The *j*th interval is interpolated by the *N^j^*-dimension Legendre polynomial $L^{j}(\tau )$; that is, the number of LGL collocations is *N^j^*. Then X, λ, β, and α can be uniformly expressed as follows:

(13) $$v^{j}(\tau )=\sum_{l=0}^{N^{j}}v_{l}^{j}\frac{\left(\tau^{2}-1){\dot{L}}^{j}(\tau \right)}{N^{j}(N^{j}+1)(\tau -\tau_{l}^{j})L_{l}^{j}}$$

where v represents any variable in X, λ, β, and α. 

Equation (14) can be obtained by differential solving for the state expression.

(14) $$\frac{dX_{k}^{j}(\tau )}{d\tau }=\sum_{l=0}^{N^{j}}X_{l}^{j}D_{kl}^{j}$$

where $D_{kl}^{j}={\dot{L}}_{l}^{j}(\tau_{k}^{j})$ is the pseudospectral differential matrix in the *j*th interval.

Since the third-generation function is only about the function of and, it can be regarded as an independent variable, and the standing value can be obtained at other interpolation points.

(15) $$\left\{ \begin{array}{l} \frac{\partial S_{0}^{j}}{\partial \lambda_{0}^{j}}=X_{0}^{j}\\ \frac{\partial S_{m}^{j}}{\partial \lambda_{m}^{j}}=0,\;m=1,2,\cdots ,N^{j}\\ \frac{\partial S_{m}^{j}}{\partial X_{m}^{j}}=0,\;m=0,1,\cdots ,N^{j}-1\\ \frac{\partial S_{N^{j}}^{j}}{\partial X_{N^{j}}^{j}}=\lambda_{N^{j}}^{j} \end{array}\right.$$

Then, Equation (16) can be obtained.

(16) $$K^{j}\sigma^{j}+\xi^{j}\beta^{j}+\gamma^{j}=r^{j}$$

According to the control equation, U=g(X,λ,β), the boundary constraint equation can be further summarized as:

(17) $$C^{j}X^{j}-H^{j}\lambda^{j}-M^{j}\beta^{j}+V^{j}+\alpha^{j}=0$$

Then, Equation (18) can be obtained by further collation:

(18) $$\left\{ \begin{array}{l} K^{j}\sigma^{j}+\xi^{j}\beta^{j}+\gamma^{j}=r^{j}\\ \Gamma^{j}\sigma^{j}-M^{j}\beta^{j}+{\hat{V}}^{j}+\alpha^{j}=0\\ {\left(\alpha^{j}\right)}^{T}\beta^{j}=0,\alpha^{j}\geq 0,\beta^{j}\geq 0 \end{array}\right.$$

where $\Gamma^{j}=[-H^{j},H^{j}]$.

The solution form of the whole interval can be obtained by assembling the results from a single interval.

(19) $$\left\{ \begin{array}{l} K\sigma +\xi \beta +\gamma =r\\ \Gamma \sigma -M\beta +V+\alpha =0\\ \alpha^{T}\beta =0,\alpha \geq 0,\beta \geq 0 \end{array}\right.$$

where the coefficient matrix *K* is a symmetric sparse matrix.

Then, the state variables and covariate variables can be obtained.

(20) $$\sigma =-K^{-1}\xi \beta -K^{-1}(\gamma -r)$$

Thus, the solution to the optimal control problem can be obtained. Equation (21) can be obtained by substituting the solution into the boundary constraint equation.

(21) $$Y\beta +q=\alpha ,\alpha^{T}\beta =0;\alpha \geq 0,\beta \geq 0$$

where $Y=\Gamma K^{-1}\xi +M,\;q=\Gamma K^{-1}(\gamma -r)-V$.

Since α and the value of variable β are unknown in the equation, and they meet the orthogonal relationship, it can be regarded as a standard linear complementary problem. The Lemke method is used to solve the equation. The value of α and β can be obtained, and then the solutions $X^{\left[k+1\right]}$ and $U^{\left[k+1\right]}$ in this iteration are also obtained. 

**Step 4:** Whether the convergence condition $\left|(X^{\left[k+1\right]}-X^{\left[k\right]})/X^{\left[k+1\right]}\right|\leq \varepsilon$ is being satisfied (where ε is the calculation accuracy) is judged. If the convergence condition is satisfied, the iteration is stopped, and the solution in this iteration is taken as the optimal solution of the optimal control problem. On the contrary, it is set that k=k+1 to return to **Step 3**.

**Step 5:** According to the optimal solution corresponding to the optimal control problem, the Hamilton function at the end of the optimal trajectory is obtained. If it is satisfied $\left|H(t_{f})+w_{k}\right|\leq \rho$, the state and control variables and end time corresponding to the optimal trajectory in **Step 4** are the optimal solutions of Equation (7). On the contrary, the Newton iteration method shall be adopted according to the cross-sectional conditions $H(t_{f})+w_{k}=0$ of the end to obtain a new end time and return to **Step 1.**

**Figure 2:**
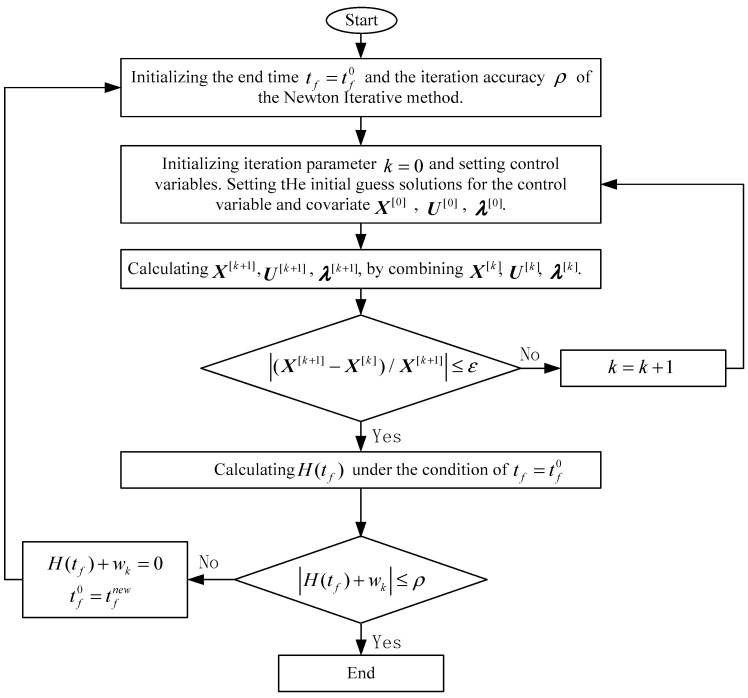
Flow chart of NSP algorithm.

The flow of the NSP algorithm is shown in Figure 2.

## 4. Numerical Simulations and Experimental Verification

### 4.1. Numerical Simulations

In order to verify the effectiveness of the algorithm, the paper uses the double-lane-change test road as the reference path for simulation analysis. The initial guesses of state and control variables at every time node are all set to be the values of state and control variables at the initial boundary; ε and ρ are set to be 10^−4^ and 10^−3^ respectively.

#### 4.1.1. A: 25 km/h

**Figure 3:**
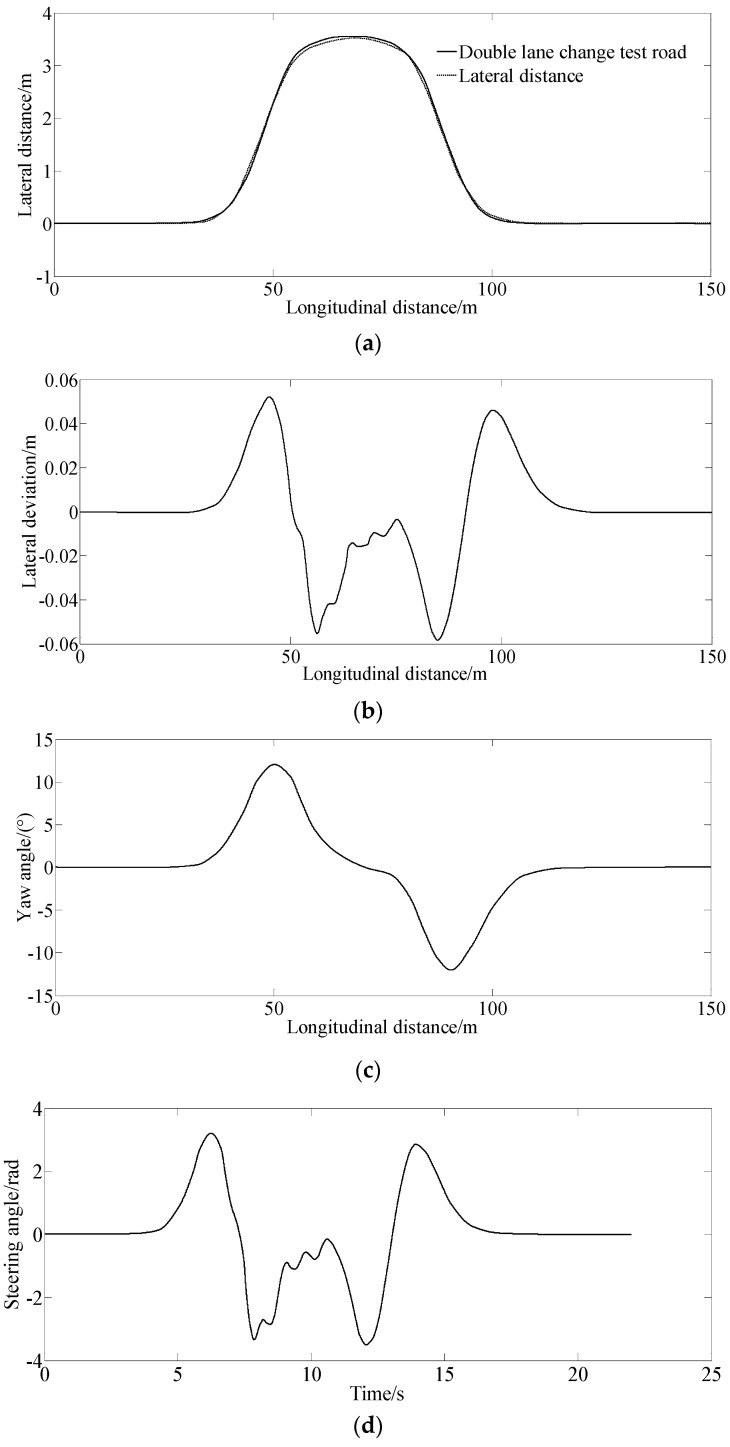

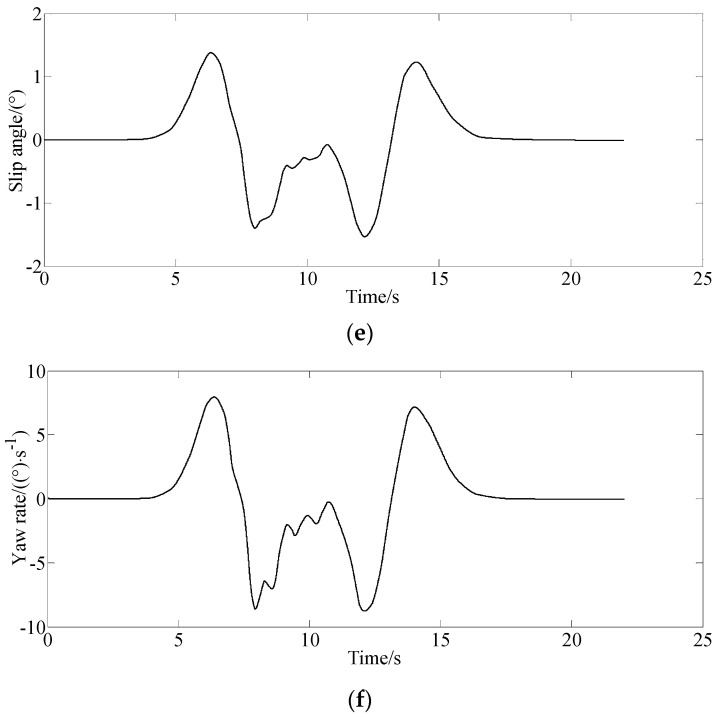
Simulation results under the condition of 25 km/h for tracking a double lane change on the test road. (**a**) Lateral distance, (**b**) Lateral deviation, (**c**) Yaw angle, (**d**) Steering angle, (**e**) Slip angle, (**f**) Yaw rate.

Figure 3 shows the simulation results under 25 km/h for tracking a double lane change road test.

Figure 3a,b show the simulation results of the lateral distance and lateral deviation. From Figure 3a,b, the lateral deviation is smaller, indicating that the vehicle can track the desired path well with the control of the proposed method.

Figure 3c indicates that the vehicle creates a larger yaw angle at the second and third corners when tracking the desired double lane change test road.

Figure 3d–f show that the vehicle needs a larger steering angle and generates a larger sideslip angle and yaw rate when entering and exiting the corners of the double lane change road test, indicating harder sledding for the driver to manipulate the vehicle and a larger degree of tension at the corners.

#### 4.1.2. B: 45 km/h

**Figure 4:**
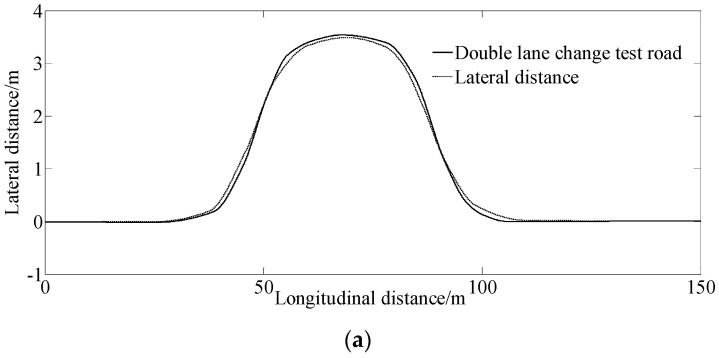

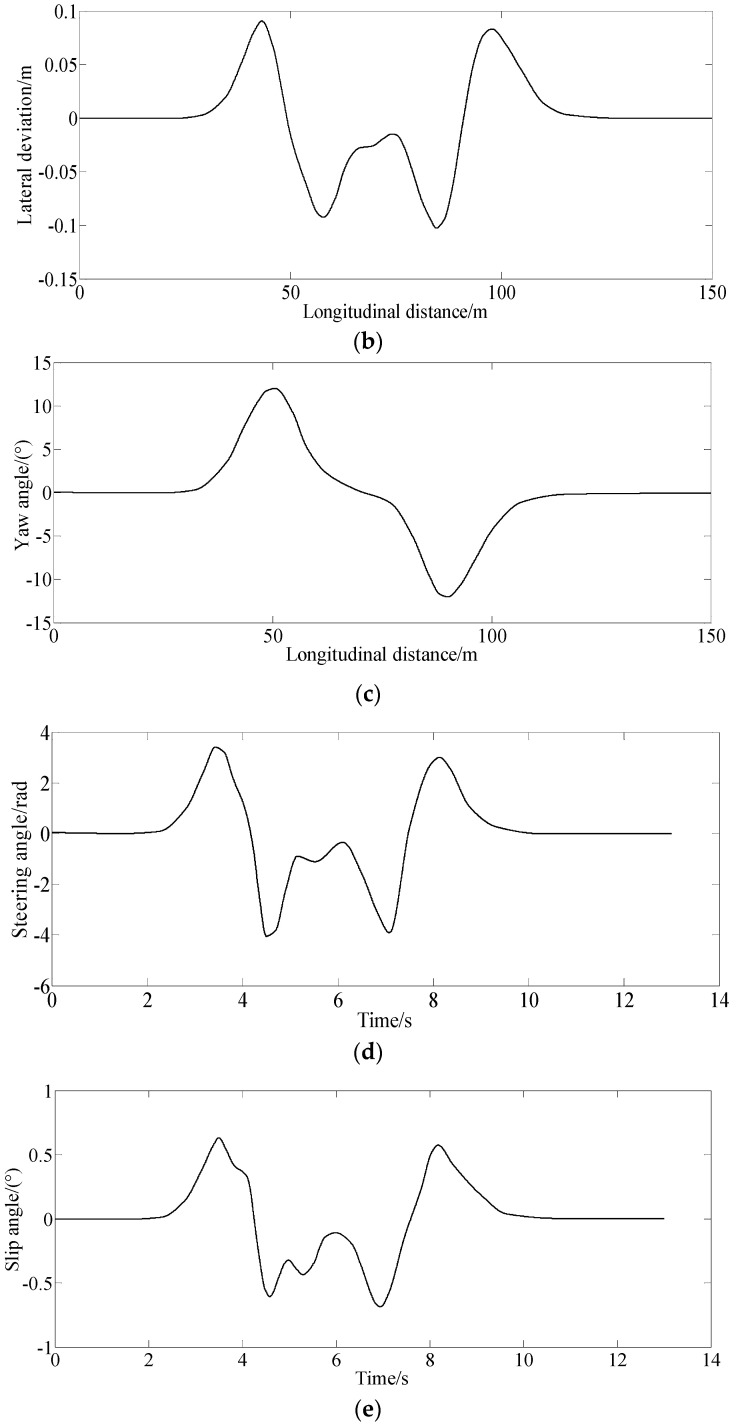

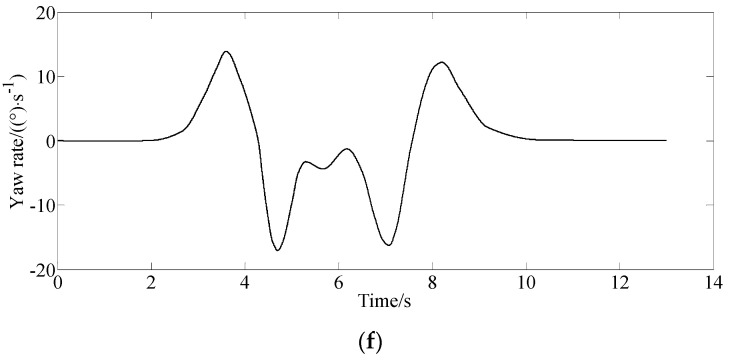
Simulation results under the condition of 45 km/h for tracking a double lane change road test. (**a**) Lateral distance, (**b**) Lateral deviation, (**c**) Yaw angle, (**d**) Steering angle, (**e**) Slip angle, (**f**) Yaw rate.

Figure 4 shows the simulation results at 45 km/h for tracking a double lane change on the test road. 

As the vehicle speed increases, the difference compared with the 25 km/h condition becomes more apparent.

Figure 4a,b shows the simulation results of the lateral distance and lateral deviation. From Figure 4a,b, we see that the vehicle can still track the desired path well with the proposed method. Figure 4c–f show that the trend of the curves is similar to that of Figure 3. However, when the vehicle enters and exits the corners of the double lane change road test, the corresponding peak values of the yaw rate, yaw angle, and steering angle were greater than at 25 km/h.

#### 4.1.3. C: 65 km/h

**Figure 5:**
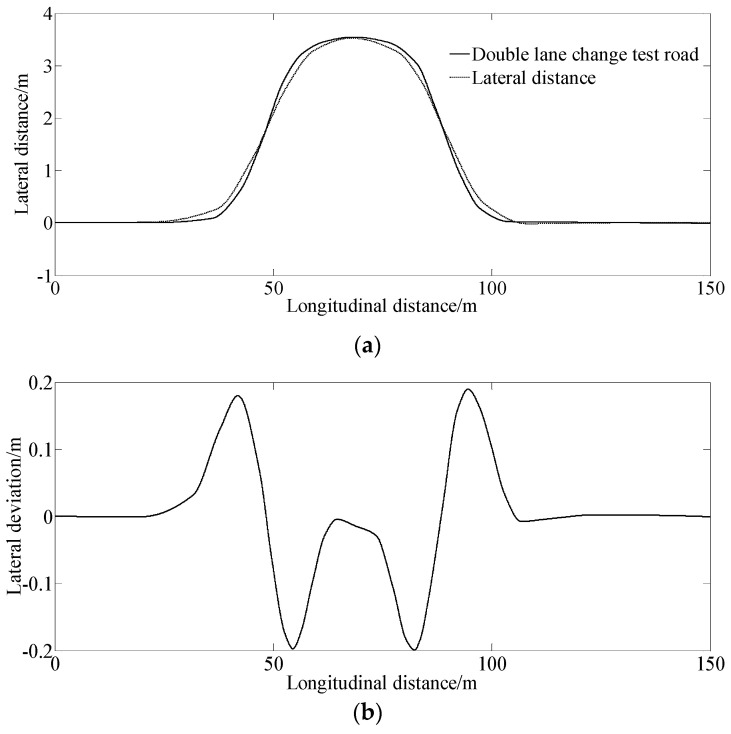

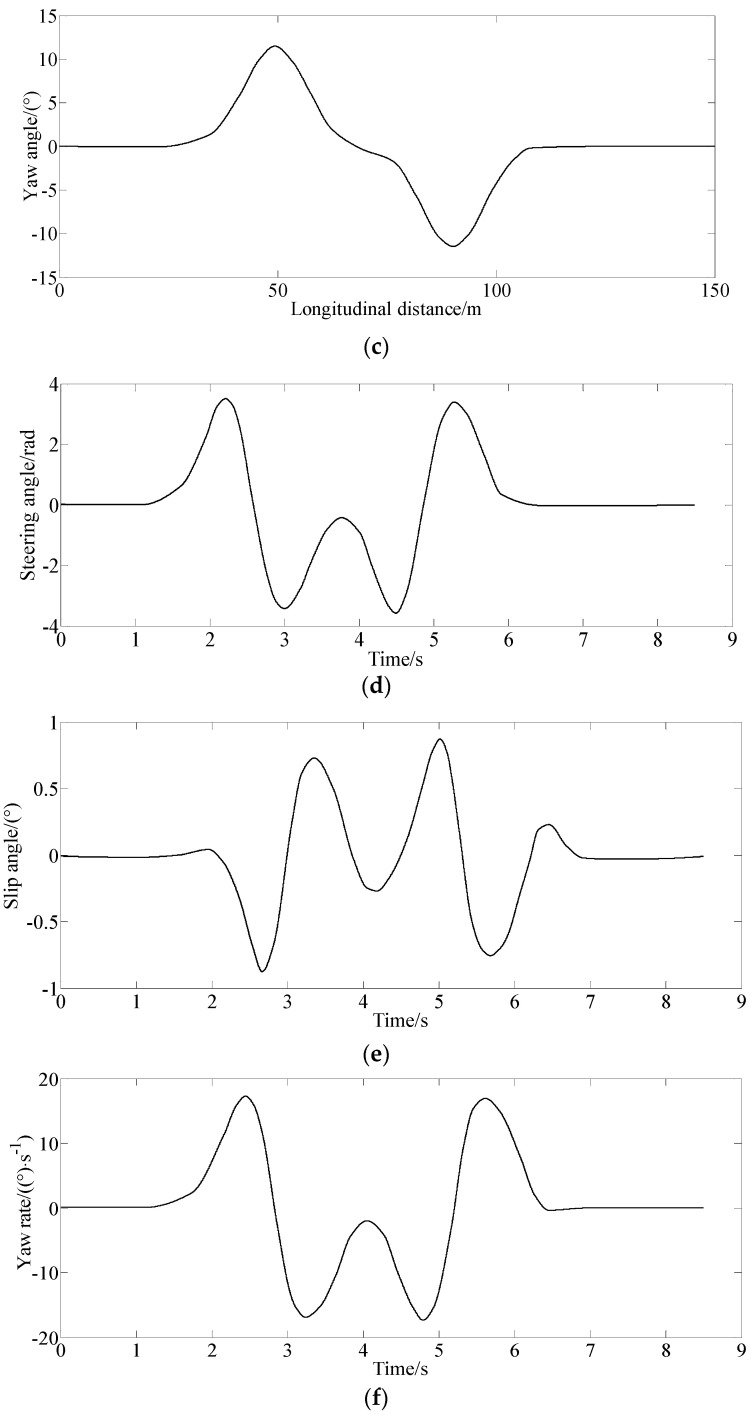
Simulation results under the condition of 65 km/h for tracking a double lane change road test. (**a**) Lateral distance, (**b**) Lateral deviation, (**c**) Yaw angle, (**d**) Steering angle, (**e**) Slip angle, (**f**) Yaw rate.

Figure 5 shows the simulation results at 65 km/h for tracking the double lane change; Figure 5a,b show the lateral distance and lateral deviation. Figure 5a,b show that the vehicle can track the desired path well with the control of the proposed method. When tracking the given path, the error between the reference and simulation results increases at higher speeds. Figure 5c–f indicate that the vehicle generates a larger yaw angle and sideslip and needs a bigger steering angle when passing the double lane change test, indicating the difficulty for the driver to manipulate the vehicle and the occurrence of the tail flick.

**Figure 6:**
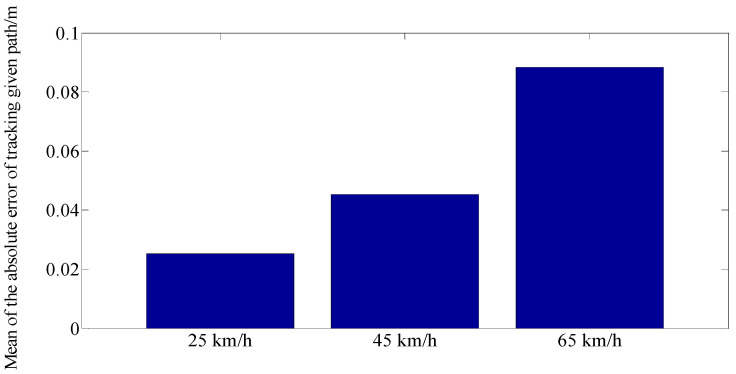
The error between the reference and simulation results at different driving speeds.

Figure 6 shows the error between the reference and simulation results at different driving speeds. As the speed increases, the error between the reference and simulation results increases. However, the rate of change from 45 km/h to 65 km/h is larger than the rate of change from 25 km/h to 45 km/h, which indicates that the control performance at 45 km/h is better than at 65 km/h.

### 4.2. Efficiency Verification

**Figure 7:**
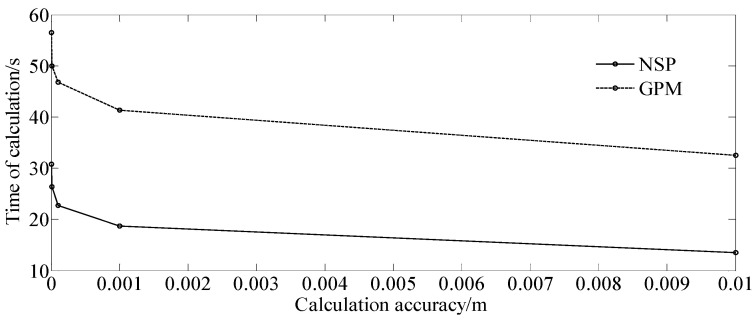
Relationship between the error and calculation time.

In order to verify the efficiency of the algorithm, we compared it with the Gauss pseudospectral method (GPM) provided in Ref. [7] under the same parameter settings. The specific comparison results are shown in Table 1, and the intuitive comparison is shown in Figure 7. It can be seen from the analysis of Table 1 and Figure 7 that, under the same calculation accuracy, the calculation efficiency of the algorithm in this paper is higher. The calculation time required by the algorithm in this paper is less than that of the Gauss pseudospectral method, which has greater advantages in calculation efficiency than that of the Gauss pseudospectral method.

**Figure 8:**
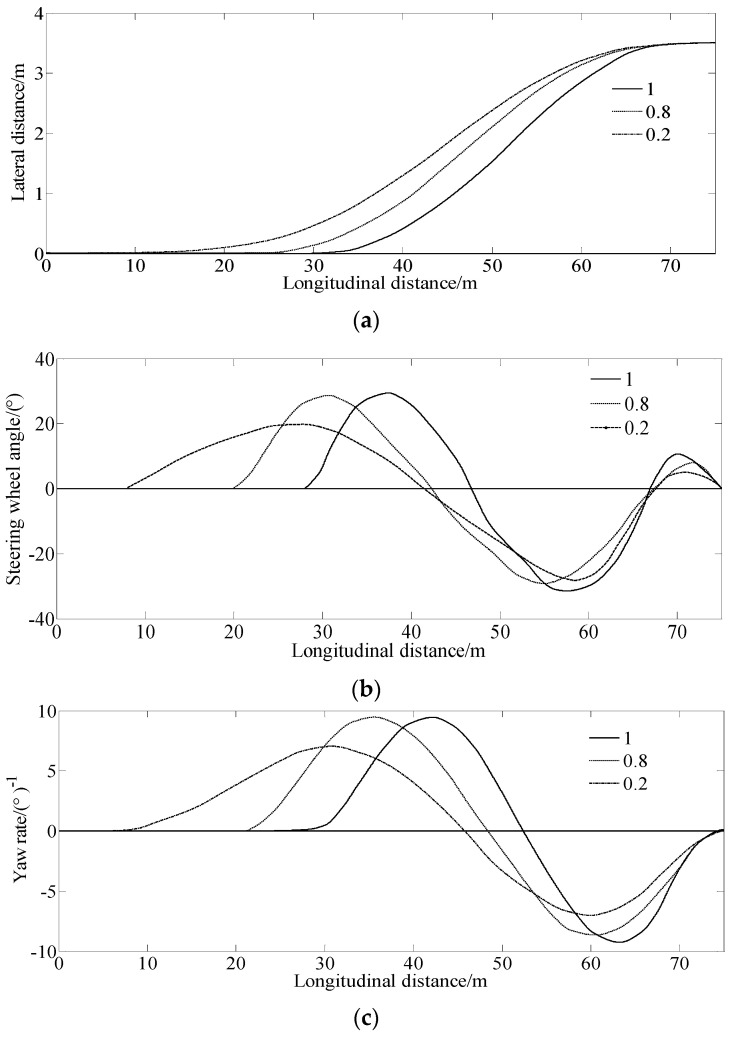
Simulation results under different road friction coefficients at u=90km/h (**a**) Lateral distance, (**b**) Steering wheel angle, (**c**) Yaw rate.

The single-lane change condition is a common scene for vehicle driving. Figure 8 shows the simulation results under different road friction coefficients at u=90km/h.

From Figure 8a, it can be seen that as the road friction coefficient decreased, the vehicle required a larger longitudinal distance to complete the single-lane change process. Figure 8b shows that the driver should establish steering options earlier to complete the single-lane change process on roads with a lower road friction coefficient. Meanwhile, Figure 8c shows that the yaw rate trend is consistent with the steering angle. As the road friction coefficient decreases, there are early occurrences of yaw rates, and the yaw rate is smaller.

### 4.3. Experimental Verification

A ground test was conducted to obtain related test data, such as the yaw rate and steering wheel angle, to verify the feasibility of the simulated results. 

**Figure 9:**
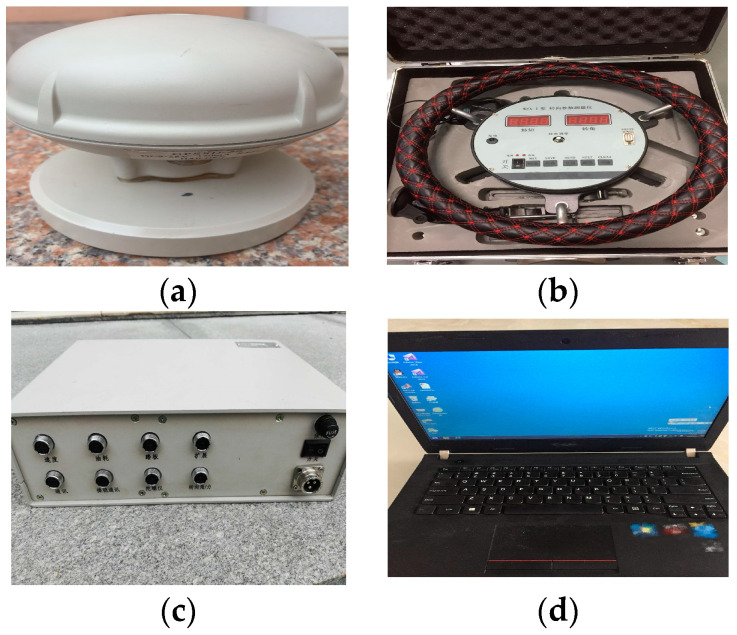
Measurement equipment. (**a**) GPSSD-20 speed instrument, (**b**) steering torque/angle tester, (**c**) AM-2800 vehicle comprehensive performance test system, and (**d**) Lenovo laptop.

**Figure 10:**
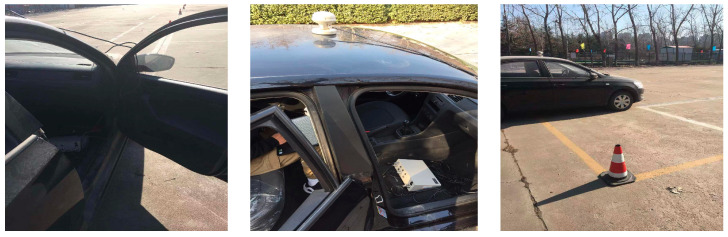
Real test vehicle.

The measurement equipment and the real test vehicle used in the tests are described in Figure 9 and Figure 10, respectively [18].

**Figure 11:**
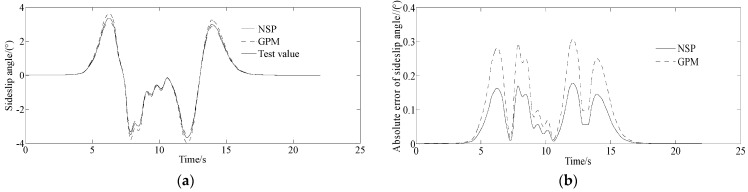

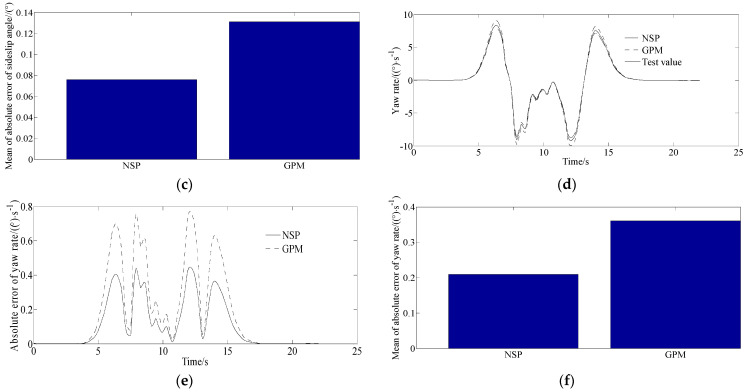
Experimental results of the sideslip angle and yaw rate. (**a**) Sideslip angle, (**b**) Absolute error of sideslip angle, (**c**) Mean of absolute error of sideslip angle, (**d**) Yaw rate, (**e**) Absolute error of yaw rate, (**f**) Mean absolute error of yaw rate.

Figure 11 shows the real vehicle test values and the numerical simulation results with the proposed algorithm. Figure 11 shows that, for the results of the sideslip angle and yaw rate, there are errors between the test and numerical simulation values. This is because the mathematical model used for numerical simulation can not fully describe the real vehicle. However, the curves in Figure 11a–f consistently illustrate the correctness of the proposed method.

The experimental results show that the algorithm proposed in this paper can solve the optimal control problem of vehicle path tracking with high accuracy, and the corresponding control variables change relatively smoothly. The algorithm has low requirements for the initial values of covariates and control variables, which has strong operability and applicability and is convenient for practical applications.

## 5. Conclusions

This article presents an optimal control method for path-tracking problems. Firstly, based on a nonlinear 7-DOF vehicle model, in view of the low computational efficiency of the traditional algorithm, the symplectic pseudospectral method based on the third-generation function of symplectic theory and pseudospectral discretization was established. Then, based on the MATLAB simulation platform, the effectiveness, and accuracy of the proposed estimation algorithm were verified under different conditions and compared with the traditional algorithm. The simulation and experiment results indicated that the mean absolute error of the sideslip angle and the mean absolute error of the yaw rate of the algorithm proposed in this paper were both smaller than the value of the GPM algorithm, indicating good calculation accuracy. And also, the mean and the standard deviation of the time of calculation of the proposed algorithm under the same calculation accuracy were both smaller than the value of the GPM algorithm, indicating a higher calculation efficiency.

In the proposed algorithm, the setting of the initial guess solution will affect the algorithm’s convergence. Improving the quality of initial guess solutions to further improve the algorithm′s convergence rate is the basis for engineering applications and the direction of subsequent research. In addition, further algorithm optimization is needed to improve its computational efficiency.

## Figures and Tables

**Table 1:** The comparison result of calculation time.

Calculation Accuracy (m)	Time of Calculation (s)			
	Mean		Standard Deviation	
	NSP	GPM	NSP	GPM
10^−2^	13.54	32.46	3.39	5.52
10^−3^	18.65	41.35	4.08	6.35
10^−4^	22.69	46.83	4.66	6.77
10^−5^	26.41	49.96	5.04	6.99
10^−6^	30.78	56.47	5.46	7.45

## Data Availability

The related data used to support the findings of this study are available from the corresponding author upon request.
